# Ramelteon combined with an α_1_-blocker decreases nocturia in men with benign prostatic hyperplasia

**DOI:** 10.1186/1471-2490-13-30

**Published:** 2013-06-12

**Authors:** Takashi Kawahara, Satoshi Morita, Hiroki Ito, Hideyuki Terao, Ryoko Sakata, Hitoshi Ishiguro, Katsuyuki Tanaka, Hiroshi Miyamoto, Junichi Matsuzaki, Yoshinobu Kubota, Hiroji Uemura

**Affiliations:** 1 Department of Urology, Ohguchi Higashi General Hospital, Yokohama, Kanagawa, Japan; 2 Department of Urology, Yokohama City University Graduate School of Medicine, 3-9, Fukuura, Kanazawa-ku, Yokohama, Kanagawa, Japan; 3 Department of Biostatistics and Epidemiology, Yokohama City University Graduate School of Medicine, Yokohama, Japan; 4 Department of Urology, Kanagawa Rehabilitation Hospital, Atsugi, Kanagawa, Japan; 5 Department of Pathology and Laboratory Medicine, University of Rochester Medical Center, Rochester, NY, USA

**Keywords:** Ramelteon, Melatonin, Nocturia, Benign prostate hyperplasia, α_1_-blocker

## Abstract

**Background:**

Nocturia is defined as waking one or more times during the night due to the urge to void. Recently, the effectiveness of several sedatives and analgesics for nocturia has been reported. We herein investigated the effects of ramelteon, an antioxidant and sleep inducer, on nocturia unresponsive to α1-blocker monotherapy in males with lower urinary tract symptoms (LUTS) as a pilot study.

**Methods:**

Subjects were 19 patients who had LUTS suggestive of benign prostate hyperplasia, received α_1_-blockers (tamsulosin, silodosin, or naftopidil), and continued to have two or more episodes of nocturia per night before starting ramelteon. Ramelteon at 8 mg once daily for one month was added to the α_1_-blocker. A self-administered questionnaire including the International Prostate Symptom Score (IPSS), quality of life (QoL) index, Overactive Bladder Symptom Score (OABSS), and Nocturia Quality-of-Life Questionnaire (N-QOL) were assessed before and one month after starting ramelteon.

**Results:**

The mean score on IPSS question 7 (nocturia) decreased significantly from 2.88 before starting ramelteon to 2.41 one month after starting the medication (*P* = 0.03). The mean total OABSS decreased significantly from 6.31 to 5.38 (*P* = 0.03), and the mean for OABSS question 2 (nighttime frequency of nocturia) also significantly decreased from 2.63 to 2.13 (*P* = 0.01). The mean total N-QOL score did not change significantly. Two patients had dizziness; the remaining patients had no adverse drug-related events.

**Conclusions:**

Ramelteon in combination with an α_1_-blocker could be a treatment option for reducing nocturia in men with BPH.

## Background

Nocturia is defined by the International Continence Society as awaking one or more times a night because of the urge to void [1]. Nocturia is known to be related to many other diseases and conditions, including overactive bladder, benign prostate hyperplasia (BPH), obstructive sleep apnea and congestive heart failure [2]. Various other factors, including nighttime liquid, caffeine and alcohol intake can also result in an increased urinary volume at night and increased nocturia [3]. The timing of therapy with diuretics, beta-blockers and xanthines may also cause some people to void more frequently at night [4]. Middelkoop *et al.* described that nocturia and associated worries were the most frequent causes of disturbed sleep in adults over the age of 50years [5]. Tsujimura *et al.* reported that urgency and nocturia are factors that independently affect sleep quality, which is closely related to patient quality of life [6]. Moreover, when nighttime waking occurs two or more times, nocturia has also been associated with a 10–21% increased risk of falling, limb fracture, excessive daytime somnolence, and nocturnal enuresis [7].

Recently, the effectiveness of some sedatives and analgesics for nocturia has been reported [8]. Drake *et al.* reported the effectiveness of melatonin for nocturia in a randomized, double-blind, placebo-controlled crossover study in 20 men [9] Shimizu et al. reported that ramelteon significantly decreased the nocturia for patients who had both LUTS and insomnia [10]. Ramelteon, the first melatonin receptor agonist approved by the United States and Japan, is a selective MT1 and MT2 agonist, [11] and its efficacy and safety for chronic insomnia has been reported [12,13]. Here we investigated the effects of ramelteon, an antioxidant and sleep inducer in humans, on nocturia unresponsive to α_1_-blocker monotherapy in men with LUTS as a pilot study.

## Methods

Subjects were 19 patients, with a median and mean (± standard deviation [SD]) age of 72.0 and 72.1±10.6 years, respectively. This study was approved by the Institutional Review Board of Ohguchi Higashi General Hospital, and written informed consent for participation was obtained from all patients. All patients with LUTS suggestive of BPH who were taking an α_1_-adrenoceptor antagonist (tamslosin, silodosin, or naftopidil) for more than 3months and experiencing two or more episodes of nocturia per night were examined before being administered ramelteon. None of the patients were taking a 5α-reductase inhibitor or anticholinergic drug. The exclusion criteria included prostate cancer, urinary tract infection, and stone disease. In this study, no patients had nocturnal polyuria or sleep disturbances. Ramelteon was taken at 8mg once daily (at bedtime) for 1month, in addition to the regular α_1_-blocker. The type of α_1_-blocker was not changed during the study period. A self-administered questionnaire including the International Prostate Symptom Score (IPSS), quality of life (QoL) index, Overactive Bladder Symptom Score (OABSS), and Nocturia Quality-of-Life Questionnaire (N-QOL) were assessed before and 1month after starting ramelteon(Additional files 1, 2, 3 and 4). The IPSS is most commonly used as an assessment tool for men with BPH or OAB, or for those who have undergone radical prostatectomy or prostatic radiotherapy [14-17]. The IPSS questionnaire comprises seven questions on LUTS (incomplete emptying, frequency, intermittency, urgency, weak stream, straining, and nocturia) and an additional question to yield a QoL index, ranging from zero (delighted) to six (terrible), which was used as the QoL surrogate indicator in this study. OAB is defined as a symptom syndrome that includes urgency, with or without urgency incontinence, and usually with frequency and nocturia [18]. N-QOL is a method for measuring treatment impact on sleep quality and on QoL thorough the evaluation of any treatment of nocturia [19].

### Statistical analysis

All continuous variables are expressed as median and mean ± SD values. Numerical data were compared using the Wilcoxon Signed Rank Sum test. An analysis of the difference in the number of patients with nocturia before and after treatment was performed using McNemar’s test. A *P*-value of 0.05 or less was considered significant. Because this study was a pilot study that evaluated a small number of patients, sample size calculations and a multivariate analysis were not performed.

## Results

Table 1 shows patient data including the type of α_1_-blocker taken and other baseline characteristics including age, prostate volume, serum prostate specific antigen (PSA) levels, and data of uroflowmetry. The data were distributed normally. In this study, no differences were noted in age, past history or medication use between the patients who were likely to respond and those who were not. There was also no significant difference in nocturia at baseline between patients taking tamsulosin silodosin and naftopidil. Five patients had hypertension with anti-hypertension drugs, three had diabetes mellitus with anti diabetes mellitus drugs, and three had a medication of a diuretic. None of the patients who were medicated with antidiabetes mellitus drugs had neurogenic conditions.

**Table 1:** Patient characteristics

**Variables**	**Number (%) or Median (mean±SD)**
No. of patients	19
Age, years	72.0 (72.1 ± 10.6)
Prostate vol, mL	37.3 (41.9 ± 25.3)
Serum PSA, ng/mL	3.6 (4.7 ± 4.1)
Uroflowmetry	
Peak flow rate, mL/s	10.5 (13.2 ± 10.0)
Average flow rate, mL/s	5.0 (7.1 ± 5.5)
Residual volume, mL	20.0 (20 ± 21.8)
Type of alpha-1-adrenergic antagonists (blockers)	
Tamsulosin (0.2 mg/day)	8 (42.1%)
Silodosin (8 mg/day)	2 (10.5%)
Naftopidil (75 mg/day)	9 (47.4%)

After 1 month of taking ramelteon, mean score on IPSS question 7 (nocturia) showed improvement, decreasing significantly from 2.88 ± 0.70 to 2.41 ± 1.00 (*P* = 0.03). However, total IPSS and QoL score did not change significantly (Table 2). Mean total OABSS also improved, decreasing significantly from 6.31 ± 2.15 to 5.38 ± 2.16 (*P* = 0.03), and likewise mean OABSS for question 2 (nighttime frequency of nocturia) decreased significantly from 2.63 ± 0.50 to 2.13 ± 0.62 (*P* = 0.01; Table 3). The mean total N-QOL score did not change significantly. However, N-QOL scores for questions 2 (Low in energy the next day) and 6 (Careful about drinking water) showed improvement, decreasing significantly from 1.60 ± 1.30 to 1.13 ± 0.99 (*P* = 0.048) and 2.20 ± 1.52 to 1.73 ± 1.28 (*P* = 0.0480), respectively (Table 4). There were no differences between the types of alpha-blockers in terms of the effectiveness against nocturia. A total of 13 patients (63.2%) exhibited a decrease in the number of nocturia episodes by more than one time. Nine (47.3%) patients exhibited nocturia one or fewer times per night. (*p* <0.01). A total of 16 patients (84.2%) wished to continue taking ramelteon because they felt that their urinary pattern was more convenient than before. On the other hand, three patients (15.8%) did not want to continue taking ramelteon because they felt that there had been no changes after adding ramelteon to their medications. Because of the small sample size in this study, we could not predict the patients who were likely to respond to ramelteon.

**Table 2:** Pre and post IPSS

**Variables**	**Median (mean±SD)**		***P *value**
	**Pre**	**Post**	
Q1	1 (1.9 ± 2.0)	1 (1.6 ± 1.7)	0.631
Q2	3 (2.8 ± 1.4)	2 (2.3 ± 1.3)	0.263
Q3	1 (1.9 ± 2.1)	1 (1.3 ± 1.3)	0.603
Q4	2 (1.8 ± 1.6)	1 (1.6 ± 1.5)	0.227
Q5	1 (1.6 ± 1.6)	1 (2.2 ± 1.9)	0.598
Q6	0 (1.1 ± 1.5)	1 (1.2 ± 1.6)	0.756
Q7	3 (2.9 ± 0.7)	2 (2.4 ± 1.0)	0.033*
total score (Q1-7)	15 (15.0 ± 7.9)	11 (12.6 ± 6.0)	0.142
QOL	5 (4.4 ± 1.5)	4 (3.8 ± 1.5)	0.206

*; *p* < 0.05.

**Table 3:** Pre and post OABSS

**Variables**	**Median (mean±SD)**		***P *value**
	**Pre**	**Post**	
Q1	1 (0.9 ± 0.3)	1 (0.8 ± 0.4)	0.083
Q2	3 (2.6 ± 0.5)	2 (2.1 ± 0.6)	0.011*
Q3	2 (2.0 ± 1.4)	1.5 (1.8 ± 1.5)	0.429
Q4	0 (0.8 ± 1.1)	0 (0.8 ± 1.2)	1.000
total score	6 (6.3 ± 2.2)	4.5 (5.4 ± 2.2)	0.027*

*; *p* < 0.05.

**Table 4:** Pre and post NQOL score

**Variables**	**Median (mean±SD)**		***P *value**
	**Pre**	**Post**	
Q1	1 (1.1 ± 1.2)	1 (1.5 ± 1.2)	0.280
Q2	2 (1.6 ± 1.3)	1 (1.1 ± 1.0)	0.048*
Q3	2 (1.3 ± 1.1)	1 (1.1 ± 1.2)	0.531
Q4	1 (1.1 ± 1.3)	1 (1.0 ± 0.8)	0.709
Q5	1 (1.6 ± 1.6)	0 (0.9 ± 1.2)	0.077
Q6	2 (2.2 ± 1.5)	2 (1.7 ± 1.3)	0.048*
Q7	2 (2.2 ± 1.4)	1 (1.6 ± 1.0)	0.070
Q8	1 (0.7 ± 0.9)	1 (1.1 ± 1.2)	0.136
Q9	1 (1.5 ± 1.5)	1 (1.4 ± 1.3)	0.806
Q10	3 (2.2 ± 1.5)	3 (2.3 ± 1.3)	0.653
Q11	3 (2.3 ± 1.5)	3 (2.4 ± 1.2)	0.634
Q12	3 (2.3 ± 1.4)	2 (2.1 ± 1.1)	0.510
Q13	4 (4.5 ± 3.1)	3 (4.1 ± 2.7)	0.604
total score	23 (24.7 ± 14.6)	23 (22.4 ± 12.3)	0.434

*; *p* < 0.05.

Two patients developed dizziness within a few days after starting ramelteon therapy; however, both patients recovered from dizziness within three to five days without discontinuing ramelteon. The remaining patients had no adverse drugs-related events.

## Discussion

We evaluated the impact of ramelteon on nocturia in patients with BPH taking an α_1_-blocker. BPH can induce bladder outlet obstruction and cause secondary bladder overactivity and a diminution of functional bladder capacity, the last two of which may result in storage symptoms including nocturia [20]. α_1_-blockers are the most common treatment for lower urinary tract symptoms (LUTS) in men because they relax prostate smooth muscle and decrease urethral resistance [21]. All currently available α_1_-blockers have similar efficacy and improve symptoms by approximately 35% and maximum urinary flow rate by 1.8–2.5 mL/s. [21] α_1_-blockers have been shown most effective in treating LUTS associated with BPH [22-24]. Nocturia is one of the LUTS, and a variety of factors can contribute to nocturia, including polyuria, nocturnal polyuria, sleep disorder, medication, bladder storage disorder (BPH, OAB, interstitial cystitis, etc.), and advanced age [25]. In addition, medical and surgical treatment for nocturia has been reported to be less satisfactory than treatment of other LUTS [8,26]. The effectiveness of hypnotic or non-steroidal anti-inflammatory drugs has been reported; however, such drugs have positional side effects including affinity for benzodiazepine which is associated with adverse effects on cognitive function with the potential for abuse, dyspepsia, and nephropathy. For ramelteon on the other hand, both safety with chronic insomnia have been reported in Japanese patients [12,13,27]. Therefore, we selected ramelteon for the BPH patients with nocturia. In this study, however, uroflowmetry findings after treatment were only checked in three patients, and no differences were found.

Nocturia is assessed in IPSS question 7, the mean score of which decreased significantly from 2.88 to 2.41 (*P* = 0.03) after participants in this study took ramelteon for 1 month. However, the impact of nocturia may not be fully captured by the IPSS questionnaire alone. Chapple *et al.* reported that the IPSS does not show how nocturia decreases sleep quality or how decreasing sleep quality affects QoL [19]. Therefore, a thorough evaluation of any treatment of nocturia requires the use of a method to measure the treatment impact on sleep quality and the QoL [19]. The International Consultation on Incontinence Questionnaire (ICIQ) N-QOL instrument, developed by Abraham *et al.*, is an easily administered, self-completed questionnaire that specifically assesses the impact of nocturia on the QoL [28]. In 2009, Yoshida *et al.* validated a Japanese version of the N-QOL. In the present study, the total score on the Japanese N-QOL did not change significantly with ramelteon, but the mean scores for questions 2 (Low in energy the next day) and 6 (Careful about drinking water) did. Question 2 might be related to the pharmacological effect of ramelteon’s association with circadian rhythm. Melatonin is currently the most accurate marker of human circadian pacemaker activity [29]. With regard to the clinical guidelines for nocturia, most patients with nocturia were coached by lifestyle guidance and behavioral therapy including “Careful about water intake”, which is the same question of N-QOL Q6 issued by urologists [25]. As a result of decreased nocturia, we speculate that patients were not concerned about drinking water.

The OABSS, originally developed in Japan, is a 4-item questionnaire to express OAB symptoms on a single scale [30]. The OABSS question items address individual symptoms: daytime frequency, nighttime frequency, urgency, and urgency incontinence. Gotoh *et al.* reported that OABSS is a useful tool for assessing the effects of treatment on OAB symptoms and responsive to treatment-related changes [31]. In the present study, total score and the score of nighttime frequency (OABSS question 2) decreased significantly (*P* = 0.01, 0.027 respectively).

Yun *et al.* reported that zolpidem combined with an α_1_-blocker was associated with decreased nocturia. Kaye *et al.* reported that oxazepam and naproxem, among five different sedatives and analgesics, significantly decreased nocturia [17]. Shimizu *et al.* reported that ramelteon decreased nocturia in a cohort of 49 patients, including patients with insomnia with prostate cancer and BPH, and those who had undergone radical prostatectomy [10]. The number of nocturia episodes was decreased from 3.1 to 2.2 per night in that study (P < 0.001). Our results support these data. The authors of that study also showed that ramelteon increased the nighttime bladder capacity from 181.4 to 201.1 cc (P < 0.05) [10]. A randomized study to confirm the effectiveness of ramelteon is needed. In the Japanese general population, the prevalence of insomnia was calculated to be about 20% and 30% in individuals aged 60 years and older [32,33]. Among widely used drugs, benzodiazepine receptor agonists exert their hypnotic effects through binding to the gamma-aminobutyric acid (GABA)-a receptor complex in the brain [34]. However, this mechanism of action of the benzodiazepines can lead to impairments in cognitive and psychomotor functions and increase the potential for dependence and withdrawal symptoms, including rebound insomnia [34]. Zopiclone also binds to GABAa receptors but differs from the benzodiazepines in the subtype of receptor it binds [25,26]. Moreover, it has a similar efficacy but is associated less frequently with next-day sedation and carries a lower risk of dependence [34,35].

Melatonin is a neurohormone secreted by the pineal gland that is involved in regulating the sleep-wake cycle and circadian rhythm [36]. Drake *et al.* reported the effectiveness of melatonin for nocturia in a randomized, double-blind, placebo-controlled crossover study in 20 men [9]. Its secretion is regulated by the suprachiasmatic nucleus, and the melatonin MT1 and MT2 receptors appear to be involved in sleep onset, while MT2 receptors mediate the phase-shifting effect of melatonin on circadian rhythm [12]. Ramelteon, the first melatonin receptor agonist approved for the treatment of insomnia in the United States and Japan, is an MT1 and MT2 agonist and has a 3- to 5-fold greater affinity for these receptors than melatonin [11]. However, ramelteon has no significant affinity for the MT3 receptor, which is located outside of the central nervous system and is not thought to be involved with sleep/circadian rhythms [11]. Ramelteon has no significant affinity for benzodiazepine, dopamine, or opiate receptors, sites that have been associated with adverse effects on cognitive function and carry the potential for abuse [11].

Melatonin is safe in older adults and reported adverse effects are infrequent, although little is known about long term use or drug interactions [9,37]. Documented side effects include decreases in body temperature, sedation, headache, depression, tachycardia, and pruritis. In our study, 2 of 19 (11%) participants experienced dizziness. Uchiyama *et al.* reported long-term safety and efficacy of ramelteon treatment for chronic insomnia [13].

This study had some limitations including the small sample size and the assessment over the short-term of only one dosage schedule of ramelteon. Previous reports have shown that naftopidil improves nocturia [38]. In the present study, all patients had received naftopidil. Eight patients in the tamsulosin group and two patients in the silodosin group had received naftopidil at some stage. These patients had not been satisfied with the effects of naftopidil. Therefore, they were switched to the another alpha-blocker. These findings suggest that ramelteon may be effective for patients who are resistant to naftopidil. In addition, we did not investigate bladder outflow obstruction using urodynamic study because of invasive examination. As such, this study provides a useful initial evaluation of ramelteon in combination with an α_1_-blocker for the treatment of nocturia. Because this study confirmed a lack of severe side effects of the treatment, we are now conducting a multicenter, randomized study of ramelteon for nocturia.

## Conclusions

Our results showed that ramelteon in combination with an α_1_-blocker could be a new option for reducing nocturia in men with BPH.

## Competing interest

All authors’ declare that they have no competing interests.

## Authors’ contributions

TK, HI, HT, RS, KT and JM obtained the data. TK and SM participated in designing of the study and performed the statistical analysis. TK and HU wrote the draft the manuscript. HI, HM, HU and YK analyzed the data. All authors read and approved the final manuscript.

## Pre-publication history

The pre-publication history for this paper can be accessed here:

http://www.biomedcentral.com/1471-2490/13/30/prepub

## Supplementary Material

Additional file 1 International Prostate Symptom Score (IPSS). Click here for file

Additional file 2 Overactive bladder symptom score (OABSS). Click here for file

Additional file 3 International Prostate Symptom Score (IPSS) Japanese version. Click here for file

Additional file 4 Overactive bladder symptom score (OABSS) Japanese version. Click here for file
