# Supplementary material for: Ramelteon combined with an α1-blocker decreases nocturia in men with benign prostatic hyperplasia
Source: BMC Urol. 2013 Jun 12;13:30. doi: 10.1186/1471-2490-13-30 (PMC3687682; doi:10.1186/1471-2490-13-30)
Supplement: Additional file 1 — International Prostate Symptom Score (IPSS). [file 1471-2490-13-30-S1.docx]

1. International Prostate Symptom Score (IPSS)

[14] Barry MJ , et al. J Urol 1992 ; 1 48 : 1549 –57
