# Supplementary material for: Ramelteon combined with an α1-blocker decreases nocturia in men with benign prostatic hyperplasia
Source: BMC Urol. 2013 Jun 12;13:30. doi: 10.1186/1471-2490-13-30 (PMC3687682; doi:10.1186/1471-2490-13-30)
Supplement: Additional file 2 — Overactive bladder symptom score (OABSS). [file 1471-2490-13-30-S2.docx]

2. Overactive bladder symptom score (OABSS)

[30] Homma Y, et al. Urology 2006; 68: 318-323.
